# Purification of Peroxisomes in a Self-Generated Gradient

**DOI:** 10.1100/tsw.2002.836

**Published:** 2002-06-07

**Authors:** John Graham

**Affiliations:** School of Biomolecular Sciences, Liverpool John Moores University, UK

**Keywords:** peroxisomes, light mitochondrial fraction, OptiPrep™, iodixanol, liver, self-generated gradient

## Abstract

In iodixanol, peroxisomes are the densest organelle in the light mitochondrial fraction and are therefore easily separated from the other components (lysosomes, mitochondria, etc.) in a self-generated gradient. Self-generated gradients make sample handling very easy and are highly reproducible but need to be formed in either a vertical, near-vertical, or small volume high-performance fixed-angle rotor. The resolution of the peroxisomes is far superior than that in sucrose and, unlike in PercollⓇ there is no contamination from endoplasmic reticulum.

**METHOD TYPE:** extraction, isolation, purification and separation

**SUB METHOD TYPE:** centrifugation

## INTRODUCTION

Peroxisomes can be purified in self-generated iodixanol gradients in high yield (80–90%) with no detectable contamination from any other organelle.

In iodixanol, peroxisomes are the densest of the major subcellular organelles (ρ = 1.17–1.120 g/ml) present in the light mitochondrial fraction from mammalian tissues and cells. Mitochondria have a median density of approx. 1.145 g/ml and lysosomes approx. 1.115 g/ml. Metrizamide or Nycodenz® gradients have been used previously to purify peroxisomes[1,2] but because mitochondria have a higher density in Nycodenz® or metrizamide than in iodixanol, the resolution of these two organelles is much easier in iodixanol gradients[3,4,5]. In Percoll, both peroxisomes and endoplasmic reticulum (ER) have the same banding density and these two organelles cannot be resolved; in iodixanol the ER has a much lower density.

The protocol below is for mammalian liver, but should be broadly applicable to other tissues and cells. For alternative methods using a preformed continuous gradient or a density barrier see Refs. [6,7], respectively.

## MATERIALS AND EQUIPMENT

OptiPrep™
Homogenization Medium (HM): 0.25 M sucrose, 1 mM EDTA, 0.1% (v/v) ethanol, 10 mM Mops-NaOH, pH 7.4.
Dilution Medium: 0.25 M sucrose, 6 mM EDTA, 0.6% (v/v) ethanol, 60 mM Mops-NaOH, pH 7.4
Working Solution (WS) of 50% iodixanol ($\rho$ = 1.272 g/ml): mix 5 vol. of OptiPrep™ with 1 vol. Dilution Medium (see Note 1).
High-speed centrifuge with a fixed-angle rotor (30–50 ml tubes)
Ultracentrifuge with a vertical, near-vertical rotor or a fixed-angle rotor (<24°) with a tube capacity of approx. 10 ml, capable of >180,000$g_{av}$ (see Note 2)
Potter-Elvehjem homogenizer (30–40 ml), clearance approx. 0.08 mm
Wall-mounted, high-torque, thyristor-controlled electric motor
Dounce homogenizer (10 ml, loose-fitting, Wheaton Type B)
Gradient unloader (tube puncture)
Syringe and metal cannula (optional)

## METHOD

Carry out all operations at 0–4°C.

1. Mince the liver very finely with scissors and transfer to a Potter-Elvehjem (Teflon and glass) homogenizer with HM (use 10 ml medium for every 2.5 g tissue). Homogenize using approx. 6 strokes of the pestle (see Note 3).
2. Centrifuge at 3000$g_{av}$ in a fixed-angle rotor for 10 min to pellet the nuclei and heavy mitochondria. This pellet may be rehomogenized in HM and the centrifugation repeated (see Note 4).
3. Centrifuge the supernatant(s) at 17,000$g_{av}$ for 10–15 min to produce a "light mitochondrial pellet".
4. Resuspend this pellet in HM using a loose-fitting Dounce homogenizer (2–3 strokes of the pestle). Adjust to a volume of about 15 ml/10 g tissue; then mix with an equal volume of WS (final iodixanol concentration = 25%: $\rho$ = 1.150 g/ml).
5. Transfer to a suitable tube (10–14 ml) for a vertical, near-vertical, or low-angle (less than 24°) fixed-angle rotor and centrifuge at a minimum of 180,000$g_{av}$. The time required for formation of the gradient will depend on the rotor type; at 180,000$g$ it will be about 3 h, at higher g-forces the time can be reduced (see Notes 5–8).
6. Allow the rotor to decelerate from 1000 rpm without the brake and collect the gradient by upward displacement or by tube puncture, or with a syringe (see Note 9).

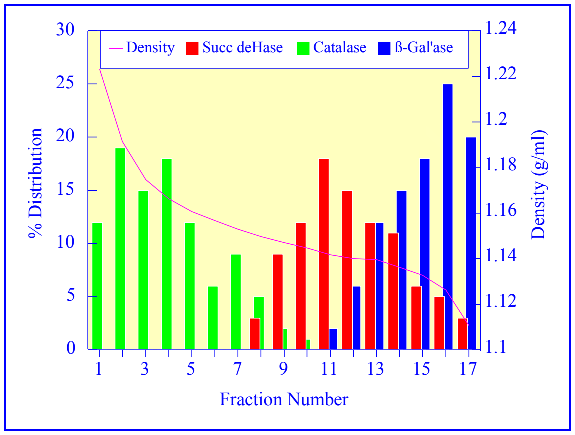

**FIGURE 1**. Isolation of peroxisomes in a self-generated gradient of iodixanol: enzyme distribution. Succ deHase = succinate dehydrogenase, ß-Gal'ase = ß-galactosidase.

## ANALYSIS

Iodixanol does not significantly inhibit any enzyme so far tested. Spectrophotometric assays carried out above 340 nm can be performed directly on gradient fractions: this includes the standard assays for catalase, succinate dehydrogenase, and ß-galactosidase[8]. Protein can also be measured directly by any Coomassie blue-based method[8]. If it is necessary to remove the gradient medium, fractions can be diluted with an equal volume of buffer; pelleted at approx 30,000$g_{av}$ for 10 min, and resuspended in a suitable buffer.

A typical result is shown in Fig. 1, which shows the distribution of marker enzymes across the gradient. The activity in each fraction is expressed as a percentage of the total in the tube before centrifugation. Fractions 1–7 contain more than 90% of the total catalase with no detectable contamination from mitochondria or lysosomes. The ER and Golgi membranes (not shown) band at the top of the gradient (far right of figure).

## NOTES

1. Strategies for preparing working solutions for mammalian tissues are given in Ref. [9]. Protease inhibitors may be included in any or all of the media at the operator's discretion.
2. The original methodology for this peroxisome purification was worked out using a fixed-angle rotor with and angle of only 20° and a tube volume of approx. 10 ml. This rotor is no longer commercially available and most fixed-angle rotors have larger angles (>24°); consequently rapid gradient self-generation is only possible with small volume high-performance rotors.
3. For more information on the homogenization of tissues and cells, see Refs. [10,11].
4. For more information on differential centrifugation of a homogenate, see Ref. [12].
5. Although much higher g-forces are required to generate the gradient than to band the organelles in a preformed gradient[8]; the use of vertical rotors, which have very short sedimentation path lengths, means that the hydrostatic pressure on the organelles is no greater than in a swinging-bucket or a fixed-angle rotor at a lower g-force.
6. The precise conditions required for peroxisome purification in self-generated gradients depend very much on the rotor type. Any vertical rotor, with a sedimentation path length of approx. 17 mm would provide a very simple and efficient system; at approx 350,000$g_{av}$ separation would take place in 1–2 h. Ref. [13] contains more information about self-generated gradients.

7. Optimal separation of the mitochondria and peroxisomes depends on the formation of a relatively shallow gradient in the middle of the tube (see Fig. 1) and a sharp gradient at the bottom to prevent the peroxisomes from hitting the wall of the tube.
8. Always check the gradient density profile that is generated in a particular rotor using a blank gradient before using it for any fractionation and adjust the centrifugation conditions as appropriate. The density of the gradient fractions may be determined by refractive index and Ref. [9] gives data for converting refractive index values to density.
9. Once the banding position of the peroxisomes has been established, the reproducibility of self-generated gradients is so high that a syringe can be used to harvest a standard volume from the bottom of the gradient. See Ref. [14] for more information on the harvesting of density gradients.

## ACKNOWLEDGEMENTS

The author and TheScientificWorld wish to thank Axis-Shield PoC, AS, Oslo, Norway for their kind permission to adapt OptiPrep™ Application Sheet S11 in the preparation of this Protocol Article.
